# Influence of sample size and analytic approach on stability and interpretation of brain‐behavior correlations in task‐related fMRI data

**DOI:** 10.1002/hbm.25217

**Published:** 2020-09-30

**Authors:** Cheryl L. Grady, Jenny R. Rieck, Daniel Nichol, Karen M. Rodrigue, Kristen M. Kennedy

**Affiliations:** ^1^ Rotman Research Institute at Baycrest Toronto Ontario Canada; ^2^ Departments of Psychiatry and Psychology University of Toronto Toronto Ontario Canada; ^3^ Center for Vital Longevity, School of Behavioral and Brain Sciences The University of Texas at Dallas Dallas Texas USA

**Keywords:** correlations, fMRI, RRID:SCR_001622, RRID:SCR_001847, RRID:SCR_002823, RRID:SCR_005990, RRID:SCR_007037, RRID:SCR_008750, sample size, statistical power, working memory

## Abstract

Limited statistical power due to small sample sizes is a problem in fMRI research. Most of the work to date has examined the impact of sample size on task‐related activation, with less attention paid to the influence of sample size on brain‐behavior correlations, especially in actual experimental fMRI data. We addressed this issue using two large data sets (a working memory task, *N* = 171, and a relational processing task, *N* = 865) and both univariate and multivariate approaches to voxel‐wise correlations. We created subsamples of different sizes and calculated correlations between task‐related activity at each voxel and task performance. Across both data sets the magnitude of the brain‐behavior correlations decreased and similarity across spatial maps increased with larger sample sizes. The multivariate technique identified more extensive correlated areas and more similarity across spatial maps, suggesting that a multivariate approach would provide a consistent advantage over univariate approaches in the stability of brain‐behavior correlations. In addition, the multivariate analyses showed that a sample size of roughly 80 or more participants would be needed for stable estimates of correlation magnitude in these data sets. Importantly, a number of additional factors would likely influence the choice of sample size for assessing such correlations in any given experiment, including the cognitive task of interest and the amount of data collected per participant. Our results provide novel experimental evidence in two independent data sets that the sample size commonly used in fMRI studies of 20–30 participants is very unlikely to be sufficient for obtaining reproducible brain‐behavior correlations, regardless of analytic approach.

## Graphical abstract

Limited statistical power due to small sample sizes is a problem in fMRI research. Most of the work to date has examined the impact of sample size on task‐related activation, with less attention paid to the influence of sample size on brain‐behavior correlations, especially in actual experimental fMRI data. Our results provide novel experimental evidence in two independent data sets that the sample size commonly used in fMRI studies of 20–30 participants is very unlikely to be sufficient for obtaining reproducible brain‐behavior correlations, regardless of whether a univariate or multivariate approach is used.

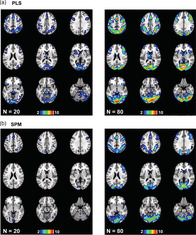

## 1 INTRODUCTION

Recently there has been discussion within the fMRI community regarding the importance of considering sample size in one's experiments. Although there is some disagreement on this topic (Friston, 2012; Ingre, 2013; Lindquist, Caffo, & Crainiceanu, 2013), it is generally accepted that limited statistical power due to small sample sizes is an issue in much of the work done in this area. Although the sample size used in fMRI studies has increased over the last two decades, the median was still below 30 in 2015 (Poldrack et al., 2017). Some very large publicly available data sets have been, and continue to be collected (e.g., Miller et al., 2016; Van Essen et al., 2013), but these are not suitable for all research questions, particularly those that require task‐based fMRI to address the neural correlates of specific cognitive processes. Hence, the need to know what sample size might be required for a given cognitive neuroscience experiment remains a critical issue (e.g., Button et al., 2013; Poldrack et al., 2017).

Several recent papers have addressed the sample size question in both resting state and task‐based fMRI studies, with highly variable results depending on the analysis method and specific brain measure assessed (Table 1). In terms of resting state studies, one found that global network efficiency assessed using data from the Human Connectome Project (HCP, van Essen et al., 2013) was reliable with 40 or more participants if the scan duration was 14 min, but 100 would be needed if the scan length was only 7 min (Termenon et al., 2016). On the other hand, when functional connectivity was extracted from resting HCP data using a number of machine learning algorithms and used to predict performance on a grip strength task, average prediction accuracy and stability appeared to plateau at sample sizes of 200 or more participants, regardless of the algorithm (Cui & Gong, 2018). The picture is even more pessimistic if group differences in functional connectivity are the focus, or if multiple scanning sites are involved. For example, a multisite study assessing differences in connectivity between healthy individuals and people with depression found that 300–400 participants in each group were needed to obtain reproducible group differences, although some brain regions would require even greater sample sizes (Xia et al., 2019).

**TABLE 1:** Summary of the literature examining the effect of sample size on fMRI results

Author (year)	Experiment type	Min # participants	Outcome measure
Termenon, Jaillard, Delon‐Martin, and Achard (2016)	Resting state FC (HCP)	40 (14 min scan)	Reproducibility of graph‐based metrics
		100 (7 min scan)	
Cui and Gong (2018)	Resting state FC (HCP)	200	Prediction of grip strength
Xia (2019)	Resting state FC (multisite)	300–400 per group	Reproducibility of group differences
Zandbelt et al. (2008)	Stop‐signal task	46 for ROIs	Task vs. control, effect size of 0.6
		100 for voxel‐wise	
Turner, Paul, Miller, and Barbey (2018)	Multiple tasks (UI and HCP)	~40 for R^2^	R^2^ or Jaccard of 0.5 (mean across tasks)
		~100 for Jaccard	
Cannon, Cao, Mathalon, Forsyth, and NAPLS Consortium (2017)	Working memory task	100 for DLPFC	Effect size of 0.5 and 90% power
		75 for sup parietal	
Desmond and Glover (2002)	Working memory (simulation)	25	Activation of 0.75% and 80% power
Thirion et al. (2007)	Button press task	27	Reproducibility (kappa) > 0.7
Cremers, Wager, and Yarkoni (2017)	Correlations (simulation)	30 (localized effect)	Reproducibility (dice coefficient) > 0.7
		> 150 (diffuse effect)	Reproducibility (dice coefficient) > 0.7
Yarkoni (2009)	Correlations (simulation)	40 (*r* = 0.7)	Sample size to reach true correlation value
		80 (*r* = 0.5)	
		> 100 (*r* = 0.3)	

Abbreviations: DLPFC, dorsolateral prefrontal cortex; FC, functional connectivity; HCP, human connectome project; Min, minimum; ROI, region of interest; Sup, superior; UI, University of Illinois.

For task‐based fMRI a similar picture emerges (Table 1). Zandbelt et al. (2008) measured test–retest replication using a stop‐signal task, and found that for a moderate effect size of 0.6 one would need 46 participants if using an ROI‐based approach, and more than twice as many participants if a voxel‐wise analysis was used. Another study showed that sample size effects depend on the task used. Across 11 tasks, correlations between derived spatial maps across samples increased up to 121 participants (the maximum number used in this study) without reaching asymptote; a mean R^2^ of 0.5 was achieved with an N of 40 participants (Turner et al., 2018). More participants were needed to reach a similar value of 0.5 when using a thresholded measure of similarity, in this case the Jaccard index. Importantly, the correlations across spatial maps were quite variable across tasks, ranging from >0.9 (language) to <0.6 (gambling) with 121 participants (for a similar reproducibility result across a different set of tasks, see Kampa et al., 2020). Furthermore, an adequate sample size may differ across brain regions. Cannon et al. (2017) showed that for all effect sizes and estimates of statistical power, one would need more participants to get reliable activation in dorsolateral prefrontal cortex during a working memory study than in the superior parietal lobe. At the lower end of the spectrum, another study (Desmond & Glover, 2002) recommended roughly 25 participants to achieve 80% power at the single voxel level for typical activation sizes and when using realistic statistical thresholds that approach those used after correcting for multiple comparisons (also see Simmons, Nelson, & Simonsohn, 2011; Thirion et al., 2007). On the other hand, rather poor overlap in activated voxels has been shown with sample sizes of 20–30, compared with a larger sample size of 58, although this was driven by false negatives, rather than false positives (Murphy & Garavan, 2004). Thus, it clear that for both resting studies and those assessing activation during cognitive tasks there is no single answer for how many participants to include in an experiment, and that brain regions of interest and type of task will need to be considered.

Although these papers illustrate the perils of small sample sizes when assessing resting state functional connectivity or task‐related activation, a critical aspect of task‐based fMRI studies is to understand individual differences in the relation between brain activity and performance on the task. Similar issues of sample size would necessarily arise when considering correlations between task‐related brain activity and behavioral measures across participants. However, to our knowledge only one empirical study has looked at the effect of sample size on such correlations in human fMRI data (Cremers et al., 2017). This study also used simulated brain‐behavior correlations to examine effects that were strong and localized versus those that were weak and diffuse. For the strong and localized condition, power and Dice coefficient replication reached maximal levels with simulated sample sizes of 30–40, whereas for weak and diffuse correlations even sample sizes of 150 failed to reach adequate power and replication levels. With both sample sizes the size of the correlations was overestimated relative to a simulated sample size of 10,000 (an effect also shown using simulations by Yarkoni, 2009; see Table 1). The real‐world example included in this study was a correlation between activity during the theory‐of‐mind task from the HCP data set and the agreeableness score obtained from a personality trait inventory. The full sample of 485 participants showed a weak and diffuse pattern of correlations, with the maximum correlation shown by any voxel of 0.25. Subsamples of *N* = 30 showed small localized areas of correlation with much higher r values (0.6–0.7), with maximum values in quite different locations in the brain. These HCP results were consistent with the simulations and supported the idea that small sample sizes can provide results that may not be stable across subsamples and may not reflect the actual underlying “true” correlations. This inflation of correlational effect sizes has been called the “winner's curse” and has been discussed as one type of mistaken inference that can result from low power due to small sample sizes (Button et al., 2013; Yarkoni, 2009). In addition, small samples may underestimate the *p* value associated with an effect, as *p* values may increase (i.e., become less significant) with adding additional participants if the original result is a false positive (Simmons et al., 2011).

Given these issues involved with small sample sizes and the relative lack of actual experimental data available for assessing these issues in brain‐behavior correlations, we aimed to explore the influence of sample size on the stability of patterns of correlations between task‐related activation and performance during fMRI scans. We assessed the effect of sample size on the replication of brain‐behavior correlations, as well as task‐related activations, obtained from two different cognitive tasks using two independent data sets, which varied in scanning parameters and preprocessing methods. This approach reflects the wide variety in methods of data collection and preprocessing in the fMRI field. One data set was from a working memory experiment (Kennedy, Boylan, Rieck, Foster, & Rodrigue, 2017) run at the University of Texas at Dallas (referred to as the Dallas data set) and the other data set consisted of the relational task from the Human Connectome Project, or HCP (Barch et al., 2013). In both cases we correlated voxel‐wise task‐related brain activity with accuracy on the task. Another goal of this study was to compare two analytic approaches, one univariate and one multivariate, which has not been done to date in the context of sample size effects in experimental task fMRI data. This allowed us to determine whether the greater sensitivity of multivariate techniques (e.g., Lukic, Wernick, & Strother, 2002) would mitigate the influence of smaller sample sizes. Our emphasis in both approaches was on replication of spatial patterns assessed with two similarity metrics, and determining the impact of sample size on the interpretation of these brain‐behavior correlational patterns.

## 2 METHODS

### 2.1 Dallas data set

The Dallas data set (Kennedy et al., 2017) consisted of fMRI scans from 171 participants aged 20–94 years (mean age = 53.03 ± 19.13 years; 100 women; 71 men). All were deemed to be healthy and cognitively normal. All participants provided written informed consent in accord with the University of Texas at Dallas and the University of Texas Southwestern Medical Center institutional review board guidelines. During scanning participants carried out a series of working memory n‐back tasks (0‐back, 2‐back, 3‐back, or 4‐back) with digits as stimuli. During these tasks participants saw a series of digits and were instructed to respond if a digit was the same as the one seen two, three, or four trials prior, or not. The 0‐back condition served as the control task and required participants to decide whether or not each digit matched a pre‐specified target digit. Each scanning run consisted of eight blocks, including two blocks of each level of difficulty, and there were three runs in total. The 0‐back blocks were 25 s in length and the 2‐back, 3‐back, and 4‐back blocks were 50 s in length. Blocks were counterbalanced for difficulty within run.

Participants were scanned on a 3T Philips Achieva scanner equipped with a 32‐channel head coil. Blood oxygenation level dependent (BOLD) data were collected using a T2*‐weighted echo‐planar imaging sequence with 29 interleaved axial slices per volume providing full brain coverage and acquired parallel to the AC‐PC line, (64 × 64 × 29 matrix, 3.4 × 3.4 × 5 mm^3^, FOV = 220 mm^2^, TE = 30 ms, TR = 1.5 s, flip angle = 60°). High‐resolution anatomical images were also collected with a T1‐weighted MP‐RAGE sequence with 160 sagittal slices (1 × 1 × 1 mm^3^ voxel size; 256 × 204 × 160 matrix, TR =8.3 ms, TE = 3.8 ms, flip angle = 12°).

Data preprocessing was performed using SPM8 (Wellcome Department of Cognitive Neurology, London, UK, RRID:SCR_007037) along with in‐house Matlab scripts (R2012b, Mathworks, RRID:SCR_001622). Additionally, the ArtRepair toolbox in SPM (RRID:SCR_005990) was used to determine motion parameter estimates. Functional images were adjusted for slice acquisition time and motion correction (using six directions of motion‐estimates from ArtRepair included as nuisance regressors), and each participant's T1‐weighted anatomical image was used to co‐register the functional maps to standardized MNI space. The resulting normalized images were smoothed with an isotropic 8 mm FWHM Gaussian kernel. The final voxel size was 3 mm isotropic.

### 2.2 HCP data set

The relational task data from the HCP 1200 release were used as the second data set. We did not use the working memory task from the HCP data set because accuracy was near ceiling. We accessed 865 nonrelated participants, between the ages of 22 and 36 years for the analyses described here (mean age = 28.72 ± 3.74; 459 women; 406 men). The relational task was adapted from a task developed by Christoff and colleagues (Smith, Keramatian, & Christoff, 2007). The stimuli were six different shapes filled with 1 of 6 different textures. In the relational processing condition, participants were presented with two pairs of objects, with one pair at the top of the screen and the other pair at the bottom of the screen. They were told that they should first decide what dimension differed across the top pair of objects (e.g., shape or texture) and then they should decide whether the bottom pair of objects also differed along that same dimension. In the control matching condition, participants were shown two objects at the top of the screen and one object at the bottom of the screen, and a word in the middle of the screen (either “shape” or “texture”). The task was to decide whether the bottom object matched either of the top two objects on that dimension. In both tasks participants responded “yes” or “no” to each stimulus. There were two runs of these tasks, with three relational blocks (each 18 s in length), three control blocks (18 s long) and three fixation blocks (16 s long) in each run.

For the HCP data set (Barch, et al., 2013), whole‐brain EPI acquisitions were acquired with a 32‐channel head coil on a modified 3T Siemens Skyra (TR = 720 ms, TE = 33.1 ms, flip angle = 52°, BW =2,290 Hz/Px, in‐plane FOV = 208 × 180 mm, 72 slices, with a multi‐band acceleration factor of 8). Two runs of the task were acquired, one with a right‐to‐left and the other with a left‐to‐right phase encoding. Runs were concatenated and analyzed as a single time series.

Data preprocessing was completed using FSL (RRID:SCR_002823, Jenkinson, Beckmann, Behrens, Woolrich, & Smith, 2012), FreeSurfer (RRID:SCR_001847, Dale, Fischl, & Sereno, 1999) and Connectome Workbench (RRID:SCR_008750, Marcus et al., 2013). These steps included a gradient distortion correction, followed by FLIRT based motion correction, TOPUP‐based field map preprocessing using a spin echo field map, distortion correction and EPI to T1w registration, one step spline resampling to atlas space, and intensity normalization and bias field removal (for further details see Glasser et al., 2013). The resulting images were smoothed with an isotropic 8 mm FWHM Gaussian kernel and the final voxel size was 3 mm isotropic to match the smoothing and voxel size used for the Dallas data.

### 2.3 Sampling procedure

Multiple independent samples of specific sizes were randomly chosen from each data set; all subsamples of a given size contained different individuals, although participants could be re‐used for a different subsample size. In the Dallas data set we kept the age distribution similar across the multiple samples, by breaking the data set into 4 age‐groups of participants (20–35, 36–55, 56–69, 70–94 years) and selecting equal numbers from each age‐group for each subsample of the data. For this data set the sample sizes were 12 (10 subsamples of 12 participants each), 24 (7 subsamples), 36 (4 subsamples), 48 (3 subsamples), 60 (2 subsamples), 72 (2 subsamples) and 84 (2 subsamples). In the HCP data set the sample sizes were 20, 40, 60, 80 (10 subsamples for each), 100 (8 subsamples), 120 (7 subsamples), 140 (6 subsamples), 160 (5 subsamples), 210 (4 subsamples), 280 (3 subsamples), and 420 (2 subsamples).

### 2.4 Analytic approach and task activation analyses

All analyses were carried out with SPM8 and PLS (version 6.1311050, www.rotman-baycrest.on.ca/index.php?section=84). SPM uses the general linear model on voxel‐wise contrasts, with FWE corrections for multiple comparisons. PLS uses singular value decomposition to determine latent variables present in the data and determines the robust voxels contributing to the LVs in a single step, so there typically are no corrections for multiple comparisons (Krishnan, Williams, McIntosh, & Abdi, 2011). PLS also uses resampling to determine the significance of each LV and the robustness of each voxel's contribution to the spatial pattern associated with each LV. We used 1,000 permutations to determine the *p* value for each LV and 1,000 bootstrap resamplings to determine each voxel's contribution via the bootstrap ratio (*BSR*, voxel salience divided by the estimated *SE* of the salience from the bootstrap).

The first set of analyses determined the task effect for both data sets. For SPM the task contrast compared 0‐back to the 2, 3, and 4‐back conditions for the Dallas data set and the relational task to its control matching task for the HCP data set. Within‐participant first level models were calculated for both HCP and Dallas data, using block‐style boxcars that were convolved with the hemodynamic response function with onsets corresponding to the beginning of a specific condition block and offset corresponding to the duration of the block. Run‐level regressors were included for these individual‐level models in both the Dallas and HCP analyses, but individual trial level information within each block was not modeled. In addition, because of the large age range in the Dallas data set, and the known associations between age and head motion (Churchill, Raamana, Spring, & Strother, 2017; Pujol et al., 2014), the six motion parameter estimates were included as nuisance variables in the models for the Dallas data set (also see next section for further corrections for age). With PLS, we used the nonrotated option, which allowed us to enter a pre‐specified contrast to mimic the SPM analysis; that is, we contrasted the 0‐back to the 2, 3, and 4‐back blocks for the Dallas data set, and the relational task blocks to the control matching task blocks for the HCP data set (with blocks defined in the same way as for SPM). For SPM we report activation maps corresponding to a voxelwise FWE *p* < .05 (*t* ≥ 4.78), and for PLS we report voxels with *BSR* ≥ 4.0 (analogous to a *Z* score ≥ 4.0, *p* < .0001). These thresholds were chosen because they are qualitatively similar and somewhat conservative (Eklund, Nichols, & Knutsson, 2016). In addition, we focus here on the areas where the tasks of interest had greater activity than their respective control tasks (see Figure S1 for the areas of deactivation, i.e., where there was more activity in the control tasks).

### 2.5 Brain‐behavior analyses

For brain‐behavior analyses, we averaged accuracy on the 2, 3, and 4‐back conditions in the Dallas data set, and for the HCP data set we used accuracy averaged over all relational task trials. To compute the correlations between brain activity and these behavioral variables, we used the task‐related contrast images created with SPM and assessed correlations between these first‐level contrast images and accuracy on the task. In SPM this was done using the second‐level multiple regression module and in PLS this was done using the behavioral algorithm in the PET module (which allows for analysis on a single image per participant). Thus, these analyses were as similar as possible between SPM and PLS, allowing both univariate and multivariate approaches to the same question of how activation during the n‐back conditions (2, 3, and 4‐back combined, Dallas data set) and the relational task (HCP), relative to their respective control tasks, correlates with performance accuracy.

The Dallas data set had a large age range in the participants, which could be problematic because previous studies have found that older adults move more in the scanner than younger adults (Churchill et al., 2017; Pujol et al., 2014), and perform more poorly on working memory tasks (e.g., Craik, Morris, & Gick, 1990; De Luca et al., 2003; Foos, 1995; Gazzaley, Sheridan, Cooney, & D'Esposito, 2007; Hasher & Zacks, 1988; Kennedy et al., 2017). We examined whether these relations held in this data set by testing the correlations among age, accuracy and frame displacement (FD, a measure of head motion, for example, Geerligs, Tsvetanov, Cam, & Henson, 2017; Petrican & Grady, 2017; Van Dijk, Sabuncu, & Buckner, 2012) in the entire sample. Age and head motion were correlated (*r* = 0.46, *p* < .0001), and both age and FD were negatively correlated with accuracy on the n‐back tasks (age *r* = −0.48, *p* ≤ .0001; FD *r* = −0.29, *p* < .001). Because we wanted to explore the effects of sample size independent of any effect of aging, we corrected for age and head motion in the brain‐behavior analyses for the Dallas data. This was done in the PLS analyses by regressing age and mean FD from both the accuracy measure and the brain image (per voxel) for each participant and using the residual values in the brain‐behavior PLS analyses. For the SPM analyses, age and mean FD were entered as covariates of no interest.

Although the HCP data set had a much more restricted age range, there nevertheless were weak, but significant correlations among age, FD and accuracy on the relational task. The correlation between age and accuracy was −0.09 (*p* < .01), between age and FD was 0.12 (p < 0.001), and between accuracy and FD was −0.19 (*p* < .00001). Therefore, we used the same procedure to regress age and FD out of the brain and behavioral data for both PLS and SPM as described above for the Dallas data set.

We report the results of the brain‐behavior analyses in several ways. From the PLS analyses we obtained a *p* value for the latent variable identified by each analysis, as well as a correlation between the brain scores and accuracy. The brain scores indicate how much each participant expressed the brain pattern on the LV and so the correlation of brain scores and accuracy provides a measure of how well the whole‐brain pattern on the LV correlates with behavior. To assess the similarity between the spatial maps within each sample size for both SPM and PLS we calculated two metrics. The first was a Spearman correlation (*rho*) across all voxels in the maps for each possible pair in a given sample size. The second metric was the Jaccard index, which is the number of voxels in the intersection (overlap) between two thresholded spatial maps divided by the union of all above‐threshold voxels across both maps. We only report the Jaccard indices for voxels with positive correlations with accuracy, as there were very few above‐threshold negative correlations with accuracy in either data set. To calculate these Jaccard indices an uncorrected threshold of three was used for both SPM *t* maps and PLS *BSR* maps (approximate voxel‐level *p* = .003); this threshold was chosen to provide a reasonable estimate of the above threshold voxels without being too liberal.

Finally, to determine the voxels that contributed consistently to the brain‐behavior correlations as a function of sample size, we calculated penetration maps for each sample size using the spatial maps obtained from the SPM and PLS accuracy analyses for the voxels with positive correlations with behavior. These penetration maps indicate the voxels where some number of thresholded spatial maps overlap within each sample size, from a minimum of 2 to a maximum equal to the total number of samples of a given size. A threshold of 3 was used for all images entering into the penetration maps, and no clustering was done on the thresholded images prior to computing the penetration maps. Prior to calculating the penetration maps, images from both the PLS and SPM analyses were masked using a gray matter mask that also removed voxels estimated as “NA” (due to low signal in the SPM first‐level processing).

## 3 RESULTS

### 3.1 Dallas data set

**FIGURE 1:**
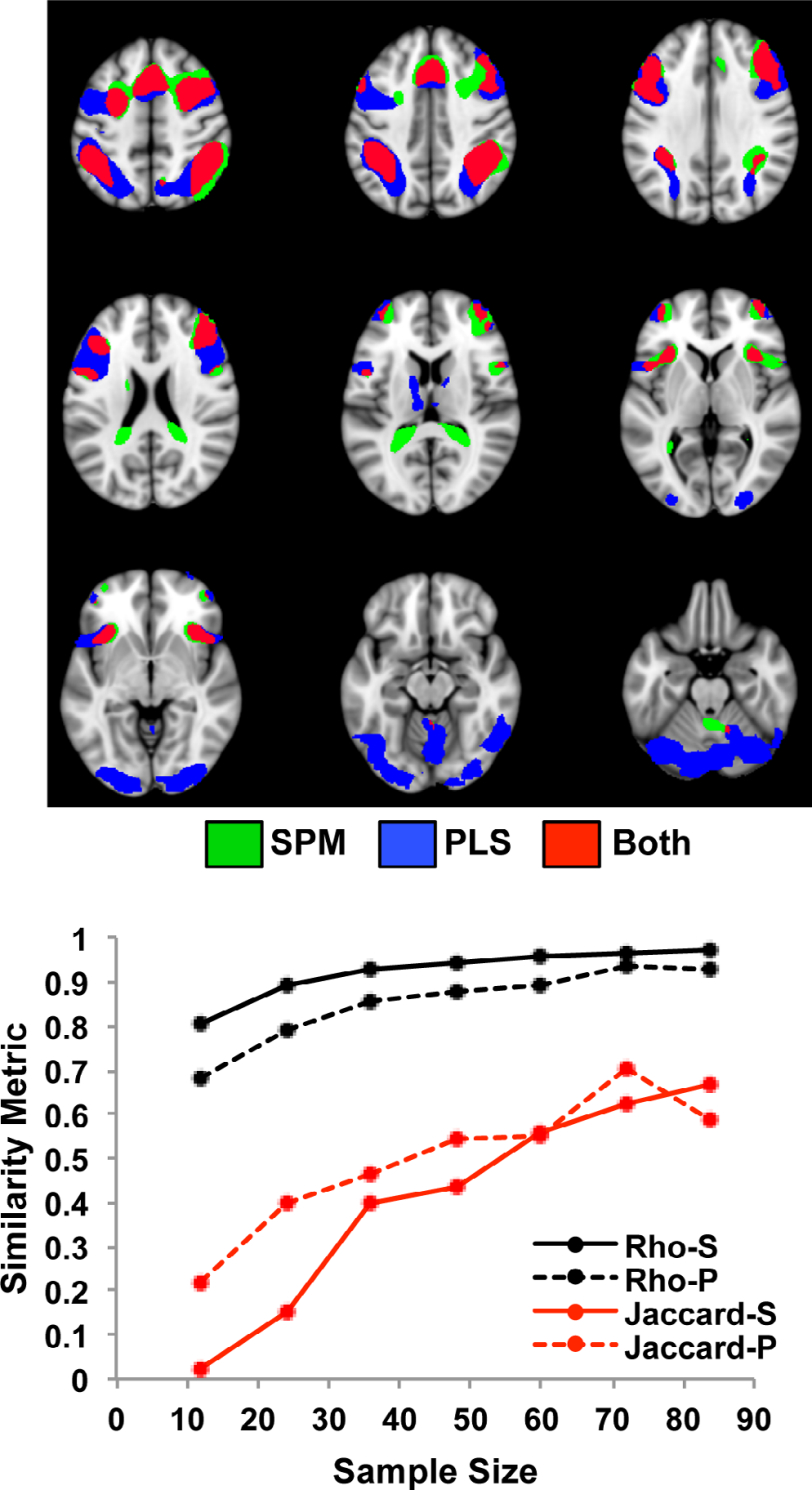
The impact of sample size on the task effect is shown for the Dallas data set using SPM and PLS. The graph shows the mean *rho* (black) and mean Jaccard values for the contrast of the 2, 3, and 4‐back working memory tasks versus the 0‐back (red), for each sample size. “S” refers to SPM, and “P” refers to PLS. The spatial maps show the mean of the two images from the subsamples with the maximum size (84 participants) where there was more activity for the 2, 3, and 4‐back working memory tasks identified by SPM (green) and PLS (blue), as well as the overlap (red). The threshold used for these maps was t > 4.7 for SPM and BSR > 4 for PLS. In this Figure and all subsequent brain figures (except Figure 4), the brain images range from 49 to −23 mm (top left to bottom right) relative to the anterior commissure—posterior commissure line, in 9 mm steps. All brain figures were made using Mango (http://ric.uthscsa.edu/mango/)

SPM identified a set of frontoparietal regions with more activity for the cognitively demanding n‐back conditions relative to the 0‐back condition. As seen in Figure 1 for the largest subsamples of 84 participants, these included dorsolateral prefrontal, inferior parietal and anterior insula/frontal opercular regions bilaterally (see Figure S1 for the areas with more activity for the 0‐back condition). The mean *rho* values for all samples sizes were quite high (≥ 0.8) even for the subsamples of only 12 participants. However, when comparing thresholded images resulting from the SPM analyses the overlap for those regions with increased activity during the working memory tasks, as indicated by the Jaccard index, was low for the smallest sample size, but increased to >0.6 at the largest sample size. PLS identified a similar group of frontoparietal regions with more activity during the n‐back conditions, but also showed more extensive activation in these regions, as well as activation in occipital cortex not found by SPM (Figure 1). The *rho* values were > 0.8 for all sample sizes, except for the smallest size of 12. The Jaccard index increased from 0.2 at the smallest sample size to ~0.6 at the larger sample sizes, and was numerically larger for PLS than for SPM, particularly with sample sizes of 50 or fewer participants. A paired *t* test comparing the Jaccard indices for PLS and SPM showed a trend for PLS to have higher Jaccard values (t[6] = 2.08, *p* = .08).

Both the task‐positive and the task‐negative activity for this data set show that univariate and multivariate approaches can sometimes result in somewhat different spatial maps. For example, SPM identified occipital regions with more activity during 0‐back, whereas PLS showed more activaton in occipital areas during 2,3,4‐back (compare Figure 1 with Figure S1). Although the PLS activation peaked in inferior occipital gyrus and peak deactivation found with SPM was in the lingual gyrus, this seemingly opposite effect in occipital cortex could have occurred because weights for each condition are allowed to vary in PLS but not in SPM. In addition, differences can be found across approaches because PLS attempts to find voxels that vary together across the whole brain, whereas SPM carries out statistics for each voxel independently.

**FIGURE 2:**
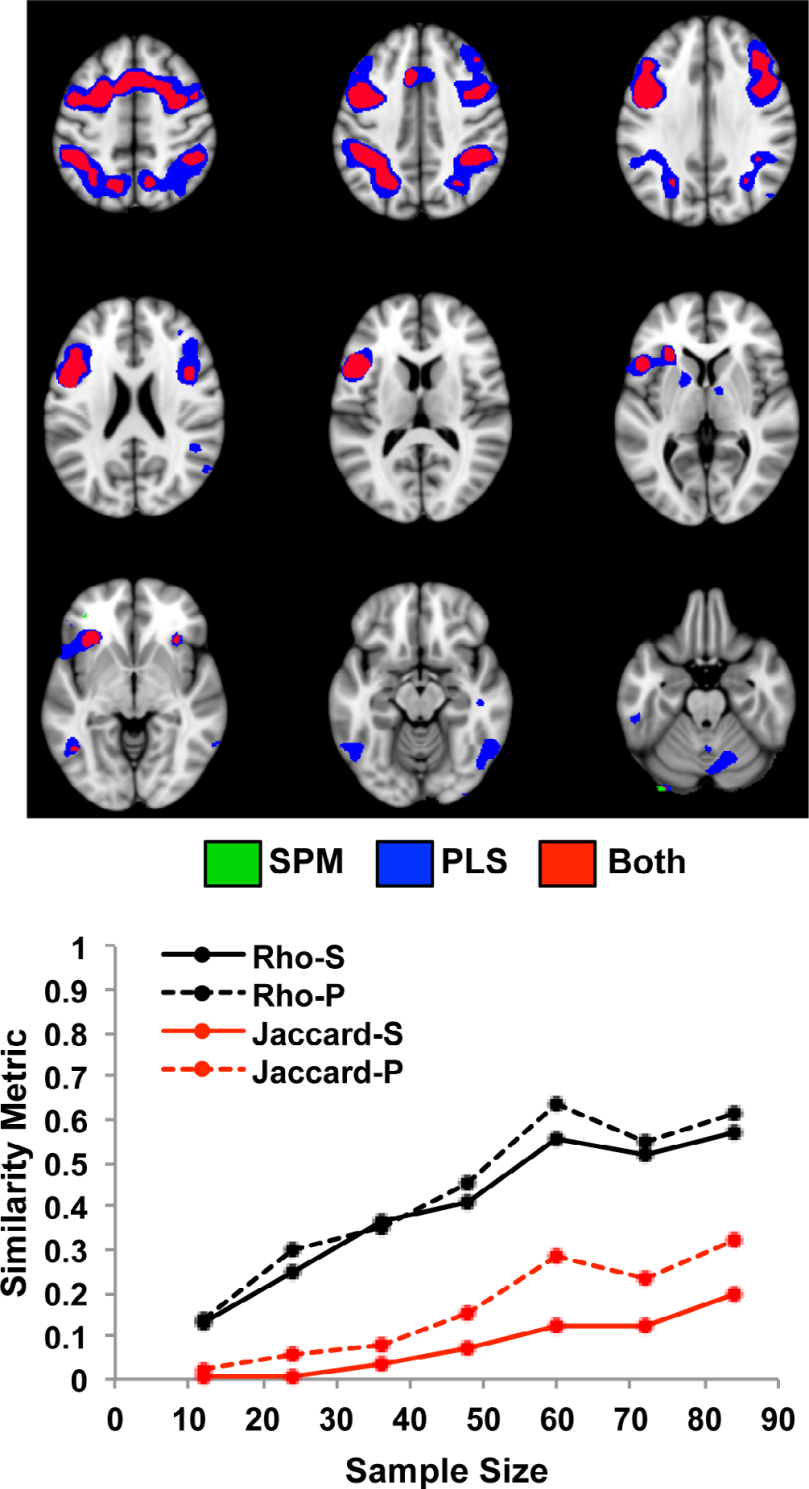
The impact of sample size on the correlations between brain activation and task accuracy is shown for the Dallas data set using SPM and PLS. The graph shows the mean *rho* (black) and the mean Jaccard values for positive correlations (red), for each sample size. “S” refers to SPM, and “P” refers to PLS. The spatial maps show the regions that were positively correlated with accuracy (mean maps for the two subsamples with 84 participants for SPM and PLS). Negative correlations were limited to only a few voxels in dorsomedial prefrontal cortex and are not shown here. The threshold used for these maps was *t* > 3 for SPM and *BSR* > 3 for PLS. Voxels identified by SPM are shown in green, those identified by PLS are shown in blue, and overlapping voxels are shown in red

As with the task activation patterns, the brain regions that correlated with accuracy on the n‐back conditions were similar for SPM and PLS (Figure 2, which shows the mean spatial patterns for the two largest subsamples). Positive correlations were seen mainly in frontoparietal cortex, with some additional regions in occipital cortex and cerebellum identified by PLS. Above threshold negative correlations were found by both SPM and PLS in only in a few very small regions in medial prefrontal cortex (not shown). The mean *rho* values increased with increasing sample size in both SPM and PLS analyses (Figure 2), peaking at 0.57 for SPM and 0.61 for PLS. The Jaccard indices for the positive correlations were considerably lower, but also increased with increasing sample size. These values were lower than the Jaccard indices for the task analyses, achieving a maximum of ~0.2–0.3, compared with ~0.6 for task. Nevertheless, the *rho* and Jaccard values were numerically higher in the PLS analyses across almost all sample sizes compared to those found with SPM. A paired *t*‐test confirmed that the Jaccard indices for PLS were significantly higher than those for SPM (t[6] = 4.49, *p* = .004).

**FIGURE 3:**
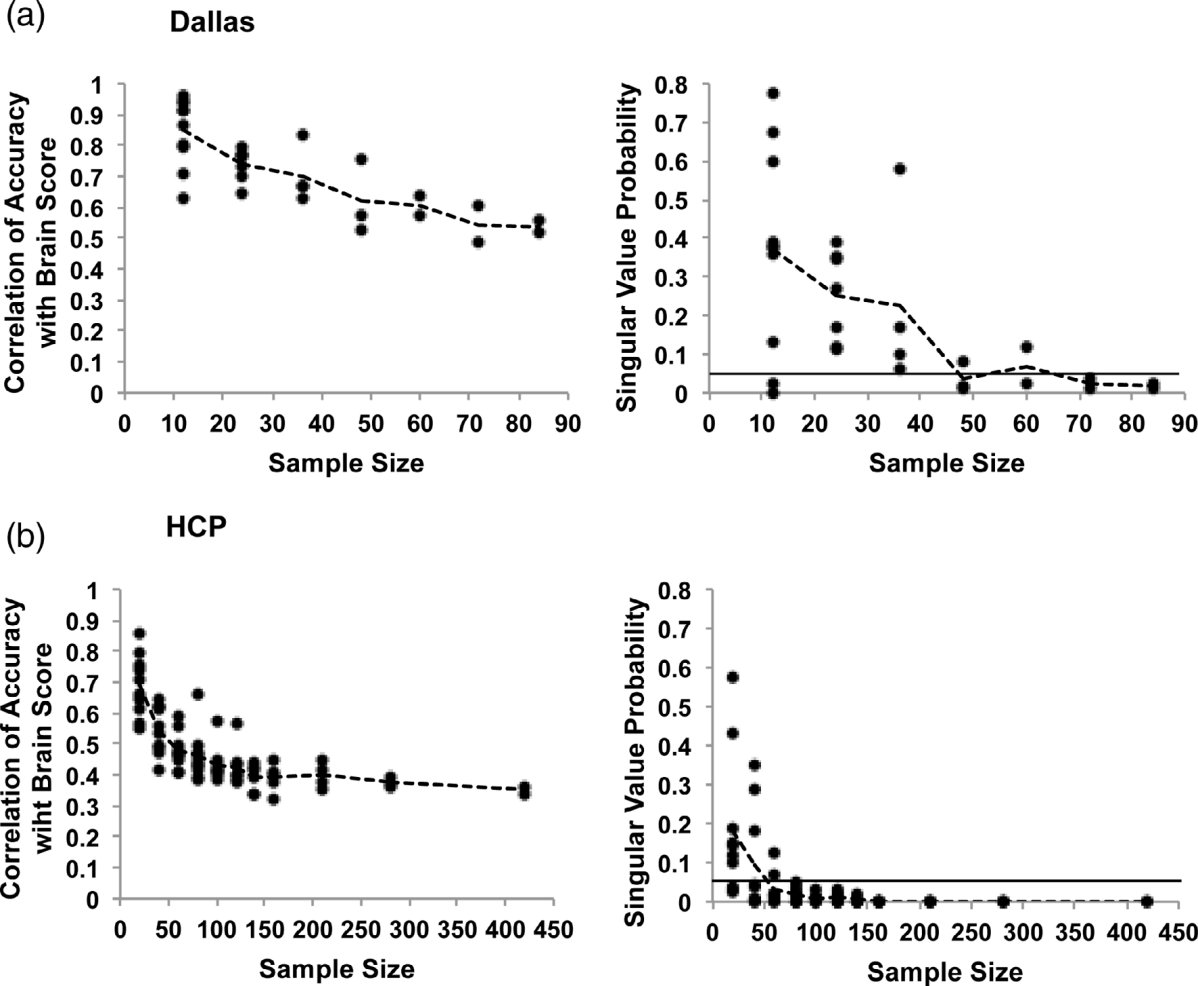
The results of the PLS analyses of brain‐behavior correlations are shown for the Dallas (a) and HCP (b) data sets. The left‐hand graphs show the correlations between accuracy and brain scores for all samples at each sample size (the dashed line joins the mean values at each sample size). The right‐hand graphs show the LV *p* values (from the permutation test) for all samples at each sample size (the dashed line joins the mean values at each sample size). The solid line indicates *p* = .05

In the PLS correlational analyses (Figure 3a), the correlations between accuracy and brain scores decreased with increasing sample size, showing the typical inflation of values with smaller sample sizes. Note, however, that all of the resulting correlations have been plotted in Figure 3 regardless of statistical significance, indicating that effect size inflation in this instance was not simply due to larger correlations being needed for significance if sample sizes are small. These correlations between accuracy and brain scores stabilized at ~0.55 with 60 or more participants. Similarly, the *p*‐values associated with the LVs decreased with increasing sample size (Figure 3a), with most *p*‐values well above .05 at the smallest sample size of 12. All the subsamples had *p*‐values below the .05 level for the two largest sample sizes of 72 and 84 participants.

**FIGURE 4:**
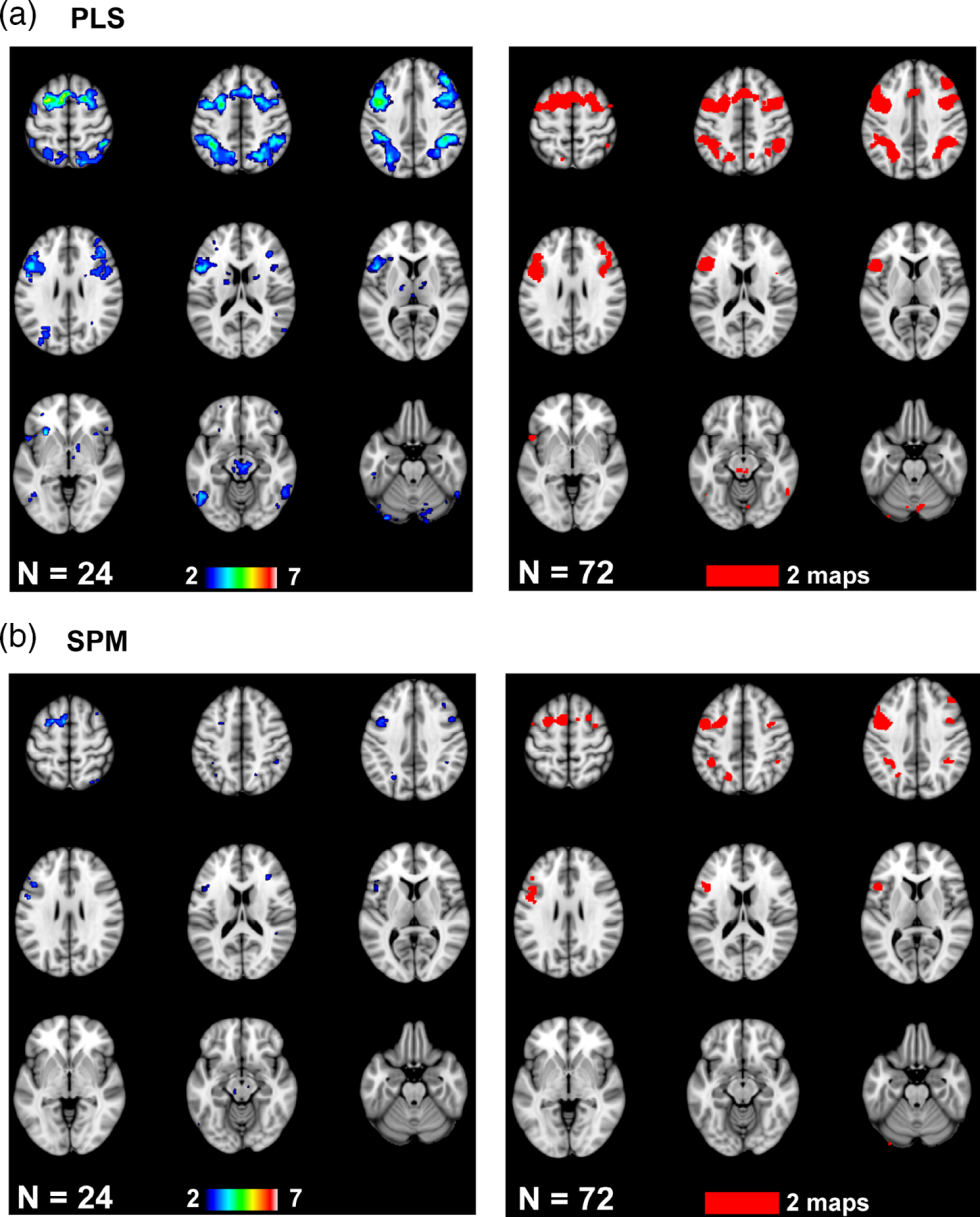
The penetration maps are shown for the Dallas data set for two sample sizes (see text for an explanation of the choice of the two sample sizes). These maps show voxels with at least two overlapping maps for PLS (a) and SPM (b). The color bars indicate the number of overlapping maps: from 2 to a maximum of 7 maps for the 24‐participant subsamples, and both of the 72‐participant subsamples (2 maps). The brain images range from 57 to −23 mm (top left to bottom right) relative to the anterior commissure—posterior commissure line, in 10 mm steps

Penetration maps for the accuracy analyses from two subsample sizes are shown in Figure 4. The sample sizes chosen for comparison in this Figure were the smallest size where all of the PLS task LVs had *p* < .05 (24 participants) and the smallest size where all of the PLS accuracy LVs had *p* < .05 (72 participants). At the relatively small sample size of 24 there was overlap across PLS maps (Figure 4a) in left precentral cortex and cerebellum (5/7 maps with overlap), bilateral inferior parietal regions and left inferior frontal cortex (4/7), and left occipital cortex (3/7). At this sample size the regions of overlap in the SPM maps (Figure 4b) were limited to small regions in left precentral cortex (3/7), bilateral parietal and frontal cortex (2/7) and left cerebellum (2/7). The regions with penetration in both PLS and SPM maps were precentral, parietal and frontal cortex in the left hemisphere. At the larger sample size of 72 the regions where the two maps overlapped in PLS were similar to those seen with 24 participants, but were more extensive in the SPM map compared to its 24‐participant counterpart. Regions common to both SPM and PLS expanded to include the majority of the regions seen in the PLS maps at both sample sizes. The main effect of increasing the sample size on the spatial patterns seemed to be to increase the number of overlapping voxels in the SPM maps without changing the basic pattern of effects. In addition, the overlapping regions identified by SPM were a subset of those from PLS, and SPM did not identify any regions not also shown by PLS. The mean number of overlapping above‐threshold voxels for the positive correlations increased as sample size increased for both SPM and PLS (Figure S2). In addition, the mean number of overlapping above‐threshold voxels identified by PLS across all sample sizes was larger than that identified by SPM (PLS mean = 1,555, *SE* = 559; SPM mean = 348, *SE* = 140; paired t[6] = 3.5, *p* = .013).

In addition to the overlap identified by the penetration maps, there also were unique regions identified by the spatial maps at the small sample size of 24 participants. Figure S3 shows all seven 24‐participant maps from the PLS analysis, which, given its greater sensitivity, would be expected to show more regions of correlation. This figure shows that all of the seven subgroups showed areas in dorsomedial or dorsolateral frontal and parietal cortex where greater activity was related to better task performance. However, a number of regions, notably in occipitotemporal cortex and subcortical regions, were identified in only one or two of the seven maps, so there was variability in these spatial patterns. In addition, there were scattered areas where reduced activity was associated with better performance (i.e., negative correlations) seen in some of the maps, and these generally did not overlap. Even in the two 84‐participant spatial maps (Figure S4) there were some areas identified in only one or the other subsample, for both SPM and PLS.

### 3.2 HCP data set

**FIGURE 5:**
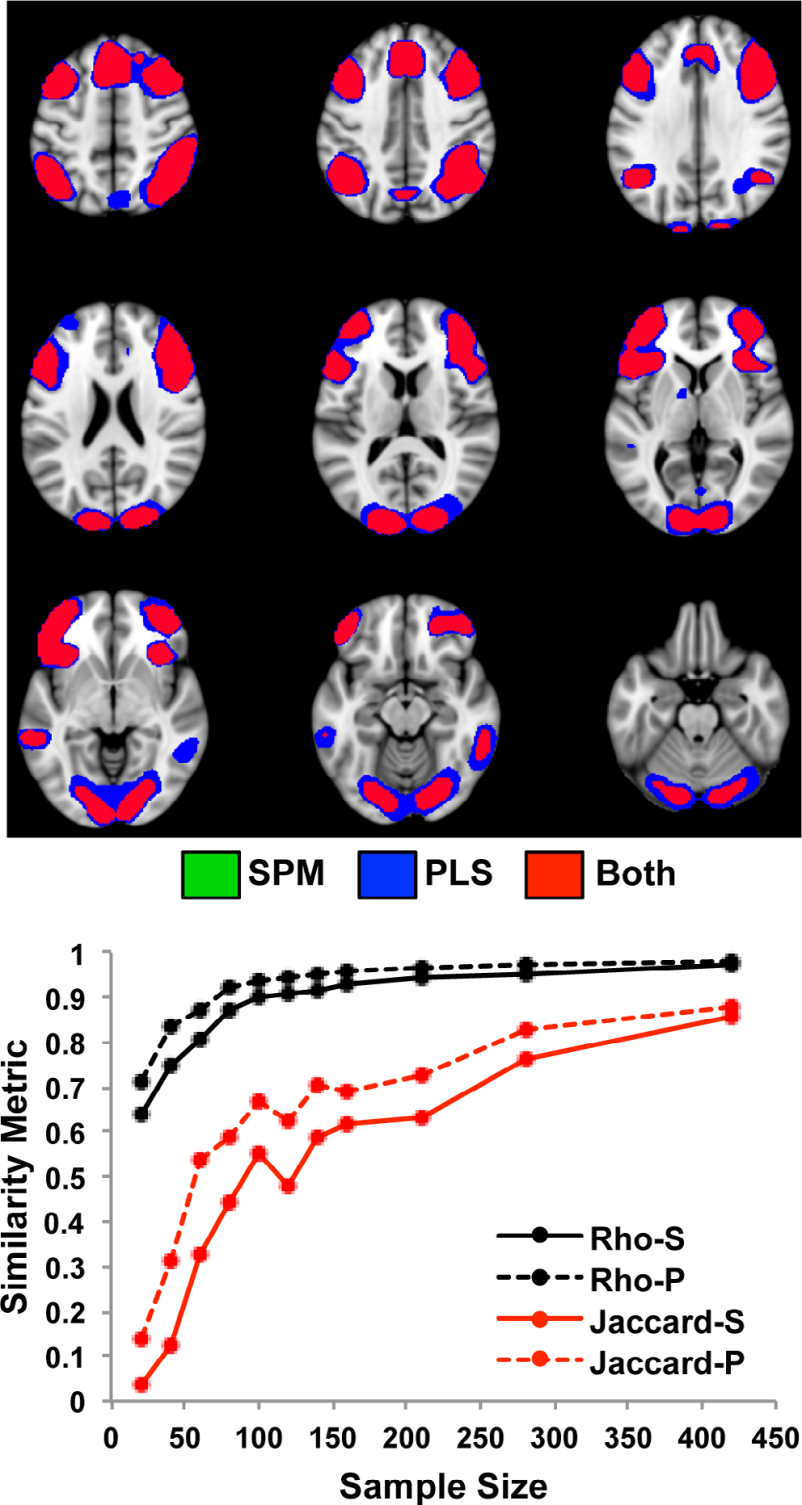
The impact of sample size on the task effect is shown for the HCP data set using SPM and PLS. The graph shows the mean rho (black) and mean Jaccard values for the contrast of relational task > control (red), for each sample size. “S” refers to SPM, and “P” refers to PLS. The spatial maps show the regions with more activity for the relational task in the samples with the maximum size (mean of the two 420‐participant subsamples for SPM and PLS). The threshold used for these maps was *t* > 5 for SPM and *BSR* > 5 for PLS. There were no voxels identified by SPM and not PLS (no green voxels) but almost all of the regions showed slightly more extensive voxels identified by PLS (blue); overlapping voxels are shown in red

For the HCP data set, the activation during the relational task revealed by SPM was in widespread areas of prefrontal and parietal cortex, and bilateral occipital cortex (Figure 5), with more activity during the control task in sensorimotor and anterior temporal regions and the cingulate gyrus (see Figure S1). This task effect identified by SPM was fairly robust even with the smallest sample size when looking at *rho*; the effect was less robust according to the Jaccard metric, which reached 0.6 only when 160 participants were included. The PLS task effect was similar (Figure 5) in terms of *rho* and Jaccard values, although the Jaccard values were higher for PLS than for SPM (paired t[10] = 8.74, *p* < .00001). Maximum Jaccard values for the relational task with 420 participants exceeded 0.8 for both SPM and PLS. The spatial maps also were quite similar but slightly more extensive in the PLS result. Overall, the relational task effect in terms of rho value was similar in strength to the working memory task effect in the Dallas data set for subsamples of similar size when using PLS (Figure S5). When using SPM the similarity metrics were consistently weaker for the HCP relational task than for the Dallas working memory task. For example, using SPM the Jaccard value at a sample size of 80, roughly equivalent to the sample size of 84 in the Dallas data set, was 0.42 for the HCP relational task and 0.67 for the working memory task in the Dallas data set.

**FIGURE 6:**
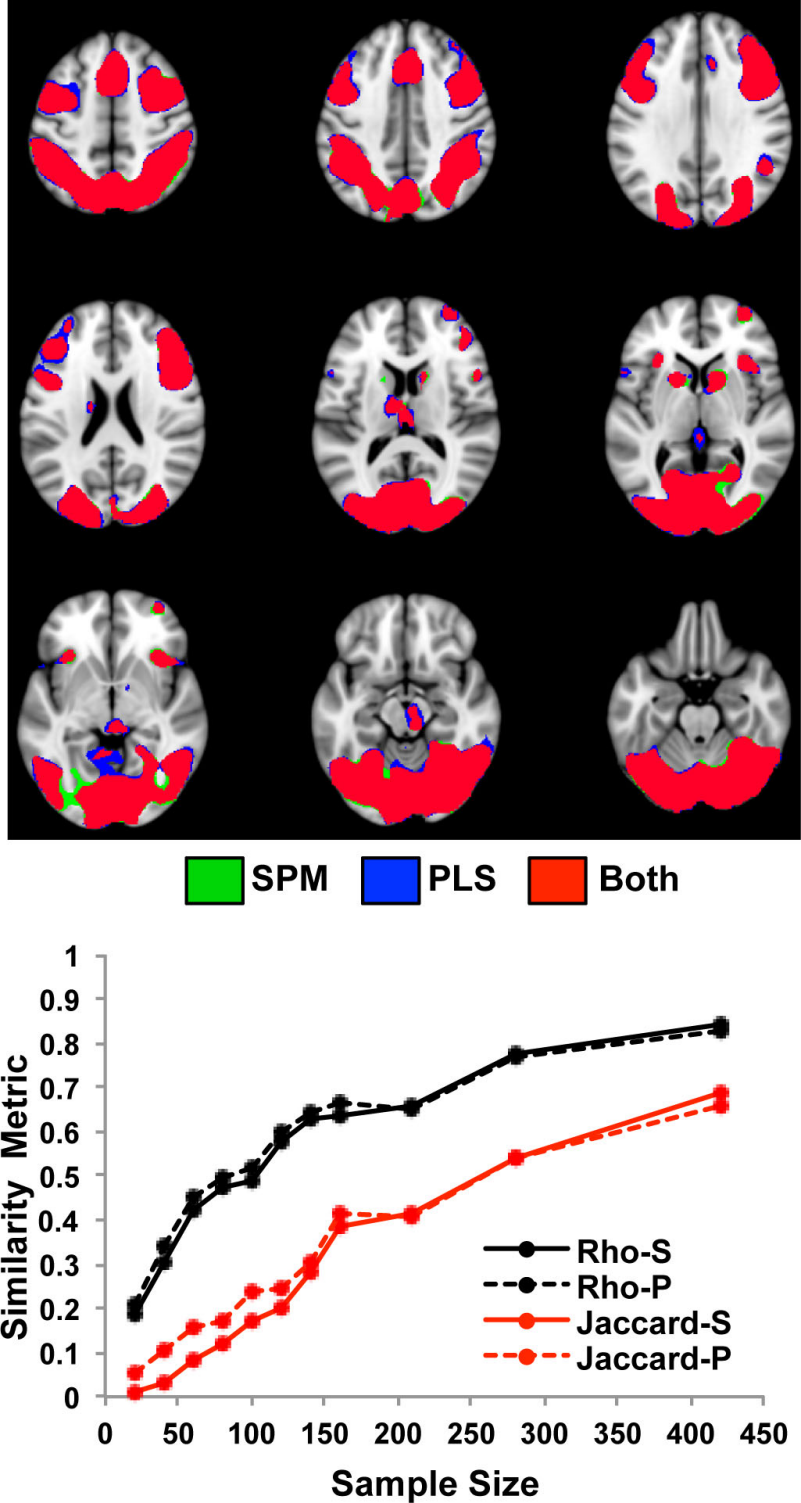
The impact of sample size on the correlations between brain activation and task accuracy is shown for the HCP data set using SPM and PLS. The graph shows the mean *rho* (black) and the mean Jaccard values for positive correlations (red), for each sample size. “S” refers to SPM, and “P” refers to PLS. The spatial maps show the regions that were positively correlated with accuracy in the samples with the maximum size (mean of the two 420‐participant subsamples for SPM and PLS). Negative correlations were limited to only a few voxels in ventromedial prefrontal cortex in the PLS analysis and are not shown here. The threshold used for these maps was *t* > 4 for SPM and *BSR* > 4 for PLS. Voxels identified by SPM are shown in green, those identified by PLS are shown in blue, and overlapping voxels are shown in red

The analyses of the relation between brain activation (relational vs. control) and accuracy on the relational task showed positive correlations in frontoparietal areas, a broad expanse of occipital cortex, and the caudate nucleus in the largest subsamples for both SPM and PLS (Figure 6). A small number of negatively correlated voxels was identified in ventromedial frontal cortex by PLS (not shown). The mean *rho* values for the analyses of relational task accuracy and brain activity increased with increasing sample size in both SPM and PLS analyses (Figure 6), peaking near 0.9, with very similar values for SPM and PLS. The Jaccard indices were lower, but also increased with increasing sample sizes in both SPM and PLS analyses, with peak values of 0.67 (SPM) and 0.76 (PLS). In addition, the Jaccard measures were higher for PLS than for SPM (paired t[10] = 2.97, *p* = .01). These *rho* and Jaccard values were similar to those from similarly sized subsamples in the Dallas data set, although the Dallas values tended to be higher, and the PLS Jaccard values were slightly higher than those resulting from SPM (Figure S5).

In the PLS analyses of the HCP data (Figure 3b), the correlations between accuracy and brain scores decreased with increasing sample size, showing inflation of values with smaller sample sizes as was seen with the Dallas data set. These correlations stabilized at ~0.4 with 100 or more participants. Similarly, the *p* values associated with the LVs decreased with increasing sample size, such that with a sample size of 80 or more the LVs from all the subsamples had *p*‐values below the .05 level. Therefore, the number of participants required to obtain relatively stable correlation magnitudes and significant LVs in the PLS accuracy analyses was roughly 80 participants in both the Dallas and HCP data sets.

**FIGURE 7:**
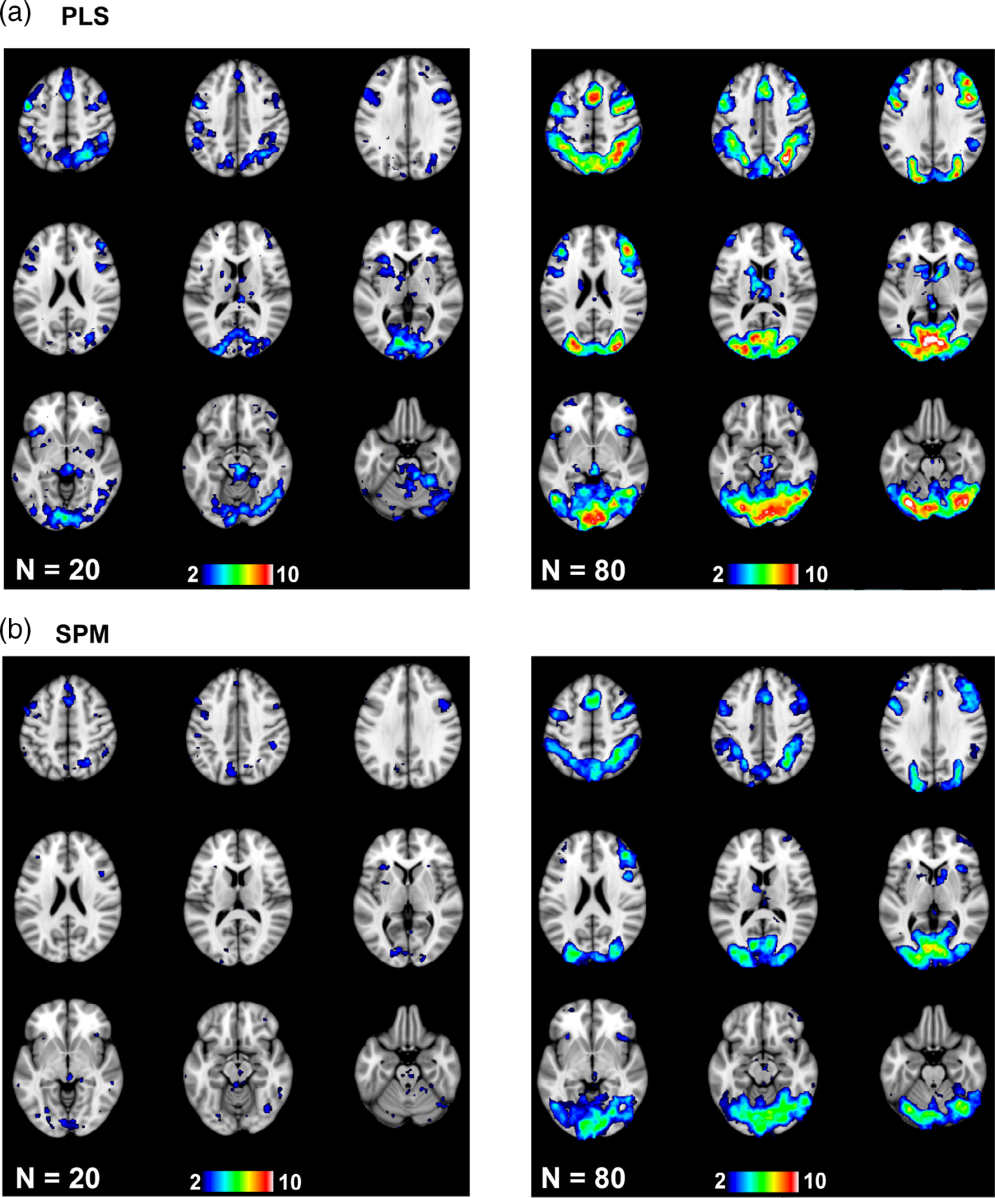
The penetration maps are shown for the HCP data set for two sample sizes (see text for an explanation of the choice of the two sample sizes). These maps show voxels with at least two overlapping maps for PLS (a) and SPM (b). The color bars indicate the number of overlapping maps: from 2 to a maximum of 10 maps for the 20‐participant subsamples, and from 2 to a maximum of 10 maps for the 80‐participant subsamples

The penetration maps for the HCP accuracy analyses (Figure 7) showed that at a sample size of 20, which was the smallest sample size where all the task PLS LVs had *p* < .05, there was overlap of positively correlated voxels in the medial occipital, parietal, and frontal regions for both SPM and PLS, although this overlap was much more extensive for PLS. In the PLS analyses a precentral region was identified in 7 of 10 of the 20‐participant subsamples, medial occipital cortex showed overlapping correlations in 6 of 10 subsamples and bilateral parietal regions had 5 of 10 subsamples with overlapping voxels. The maximum amount of overlap in the SPM maps was lower, and was found in medial occipital cortex, which showed overlap in 4 of the 10 subsamples. Right parietal and frontal cortex showed overlap in 3 maps. At a larger sample size of 80, where all the accuracy PLS LVs had *p* < .05, the occipital area was considerably larger in extent, as were the frontal and parietal cortical regions of overlap in both the PLS and SPM maps. In the PLS maps the right parietal region showed the maximum amount of overlap (10 of 10), and the frontal and medial occipital regions showed overlap in 8–9 maps. In the SPM penetration maps 8 of 10 maps overlapped in the medial occipital region and right frontal cortex, with the maximum amount of overlap (9/10) in a small region of left cerebellum. As with the Dallas data set, the mean number of overlapping voxels identified in these spatial patterns increased with increasing sample size (Figure S2). Also, PLS showed a greater number of overlapping voxels than SPM (PLS mean = 6,894, *SE* = 1876; SPM mean = 5,885, *SE* = 2024; paired t[10] = 5.0, *p* = .001). Thus, as with the Dallas data set, larger sample sizes in the HCP data were associated with a greater extent of regions reliably found across subsamples and techniques, revealing more frontal, parietal, and occipital cortex involvement. The PLS and SPM maps were similar, but the PLS maps showed more overlapping voxels.

The 10 individual maps from the 20‐participant subsamples (PLS analysis) are shown in Figure S6 to illustrate the variability across maps with this small sample size. As with the Dallas data set, there was variability in the extent of correlation in frontoparietal cortex as well as inconsistent correlations in subcortical and temporal regions. In addition, there were scattered areas of negative correlations between activation and task accuracy that generally did not overlap across maps. Finally, there was an area in left medial parietal cortex that showed positive correlations with accuracy in four subsamples, but negative correlations in two of the subsamples. In addition, there continued to be some unique positively correlated voxels in the spatial maps from the two largest HCP subsamples (420 participants, Figure S4)

## 4 DISCUSSION

As expected, all of our analyses showed an increase in the stability of the derived spatial maps as sample size increased, as well as a decrease in the size of the correlation between brain activity and task accuracy identified by PLS. These task and brain‐behavior effects appeared to be similarly robust in the Dallas and HCP data sets, with slightly greater similarity measures in the Dallas data set when using SPM to analyze task effects. Overall, our results are consistent with the literature on the disadvantages of small sample sizes and extend this work by showing in two independent data sets: (a) sample size influences brain‐behavior correlations in a similar way regardless of whether one uses a univariate or multivariate analytic approach, although the multivariate approach identified more consistent and extensive correlational patterns; and (b) the effects of small sample size on interpretation of brain‐behavior patterns include type I and type II errors, as well as inflated correlation effects.

Consistent with prior work, we found that increasing the sample size increased the similarity of the spatial maps for the task effect using both *rho* and the Jaccard index (Murphy & Garavan, 2004; Turner et al., 2018). In addition, the *rho* values calculated on the unthresholded images were consistently higher than the Jaccard values calculated on the thresholded images. For example, in the Dallas data set the amount of variance shared by the unthresholded task‐analysis spatial maps from the two largest subsamples (84 participants) was ~90% whereas the overlap of the two thresholded images was ~40%. This difference in similarity metrics calculated on task activations also was noted by Turner et al. (2018), and suggests that thresholding the images prior to assessing map similarity removes some information that contributes to assessing stability across spatial maps. Also, the *rho* and Jaccard values that we report here for both Dallas and HCP are well within the range of values reported by Turner et al., which were calculated on a much smaller sample from the HCP, thus replicating their work and indicating that these values can generalize across independent data sets.

We also showed that increasing the sample size increased the similarity of the spatial maps for the accuracy analyses, indicating that brain‐behavior correlations also benefit from greater stability of results with larger samples. In line with a prior simulation (Yarkoni, 2009) we found that brain‐behavior correlations were over‐estimated with small sample sizes but were largely stabilized with sample sizes of roughly 80 or more participants. In addition, the brain‐behavior correlations were less stable than the task effects for both data sets, particularly in terms of the Jaccard index, across all sample sizes. This is perhaps not surprising as this difference in power has been reported in prior work using simulations to estimate power (Yarkoni, 2009), although studies assessing brain‐behavior correlations typically do not utilize larger sample sizes than those assessing task effects (Lebreton, Bavard, Daunizeau, & Palminteri, 2019). Nevertheless, it is clear that more participants would be needed if the research question required an assessment of individual differences in the relation between task activity and behavior.

Like several prior simulation studies (Cremers et al., 2017; Yarkoni, 2009), we found using PLS that correlations between brain activity and accuracy were inflated with small sample sizes in both data sets. This inflation has typically been found as a result of larger r values being needed to reach a statistical cutoff with a small sample. However, these correlations derived from PLS are not assessed for significance per se, because significance is assessed at the LV level (and many of the correlations were not associated with significant LVs in the small sample sizes, see Figure 3). Therefore, the inflation of correlation values that we found with PLS is not dependent on whether or not the correlation is significant. Instead, this inflation may occur with PLS because PLS, as a multivariate technique, models the optimal relation between whole brain activity and accuracy. With small sample sizes, any such model may be over‐fitted, leading to inflated correlation values and LVs that are not stable or significant when assessed using the permutation test. With larger samples the relation between whole brain activity and accuracy can be modeled more accurately, with less over‐fitting and more stable LVs. Thus, overly large correlation values with small sample sizes can result from both univariate and multivariate analytic techniques, and are not necessarily a function of statistical thresholding.

Our results also indicate that the brain‐behavior and task effects are similarly robust across the Dallas and HCP data sets at comparable subsample sizes, with a slight advantage for the Dallas data (see Figure S5). It is important to note that the larger age range in the Dallas participants did not influence this effect because we removed the effects of age and head motion prior to calculating the brain‐behavior correlations. The slightly greater sensitivity seen in the Dallas data set could be due to several factors. The HCP scanning parameters and pre‐processing differ from those used with the Dallas data set, which could influence the results. The difference between data sets also could reflect a difference in the task demands as variability across tasks in reproducibility has been found by others as well (Kampa et al., 2020; Turner et al., 2018). In addition, there was more data per‐participant in the Dallas data set than for the HCP, and the amount of data collected for each participant is a known factor in determining statistical power in fMRI (Desmond & Glover, 2002; Mumford & Nichols, 2008; Nee, 2019). One factor that does not appear to account for differences in results across the two data sets is the range of accuracy scores. The range of these scores is similar in the two data sets (roughly between 50–100%), so the degree of behavioral variability does not seem to be a factor.

The main difference in the results between the SPM and PLS approaches to analysis was that PLS identified more voxels with either a task or correlation effect, and there were higher Jaccard values and more overlap in the penetration maps from the correlation analyses compared to SPM. This difference was consistent across both data sets, although in the HCP data set it was more prominent at smaller sample sizes (i.e., less than 200 participants). This finding is consistent with evidence that multivariate analyses can be more sensitive than univariate ones (Fletcher et al., 1996; Lukic et al., 2002), but also extends this evidence to show that multivariate assessments also can result in more consistent and stable patterns of correlational effects. Thus, our results suggest that the basic influence of sample size on results stability and the interpretation that one would gain from either a task or behavioral analysis would not depend on which approach was used. However, given the greater sensitivity of PLS one would likely require fewer participants if using a multivariate approach than if using a univariate approach.

In regard to the interpretation, there are several points of interest to note. First, the task effects that we found replicate the regional effects reported by others on these tasks. That is, the working memory task engaged regions of dorsolateral prefrontal and parietal cortex that have been reported many times by prior studies in young and older adults (for recent meta‐analyses see Daniel, Katz, & Robinson, 2016; Rottschy et al., 2012; Wang et al., 2019; Yaple, Stevens, & Arsalidou, 2019). The relational task also engaged regions reported for this task by other researchers (Barch, et al., 2013; Smith et al., 2007), such as dorsomedial and lateral prefrontal cortex, and occipital regions. In addition, both the working memory and relational task activate similar cognitive control regions in frontal and parietal cortex (e.g., Dosenbach et al., 2007; Power et al., 2011; Vincent, Kahn, Snyder, Raichle, & Buckner, 2008), indicating a demand on cognitive control despite the differences in the perceptual and memory domains. Second, for both data sets the task‐related increases in activity were positively correlated with accuracy on the tasks. In the Dallas data set greater activation in frontoparietal regions during the n‐back tasks, compared to 0‐back, was related to higher accuracy on the tasks. In the HCP data set more activation during the relational task in frontoparietal and occipital cortices was related to better performance on the relational task. These positive correlations between task‐related activity and performance are in line with the correlation between working memory load‐related activation and accuracy reported in the full Dallas data set by Kennedy et al. (2017), and the finding that activation in parietal and occipital cortex during the relational task correlated with performance on a cognitive control behavioral composite in 194 of the HPC participants (Lerman‐Sinkoff et al., 2017). Thus, for both tasks, activation in frontoparietal regions thought to be involved in cognitive control is associated with better performance. This finding is particularly notable regarding the working memory task, as working memory is thought to be one of the main components of cognitive control (Miyake et al., 2000), providing further support for the role of these specific frontal and parietal regions in top‐down control processes. Third, it is important to note that an increase in the spatial extent of voxels where activity is correlated consistently with accuracy is an important effect of increasing the sample size. That is, with smaller sample sizes the spatial patterns identified in the penetration maps were more spatially restricted than those from larger sample sizes (a similar effect of sample size on task‐related activation was noted by Murphy & Garavan, 2004). With larger sample sizes it became evident that activity in almost all of the task‐related regions was correlated with behavior, indicating a strong link between the regions engaged by the task and participants' ability to do the task accurately. This result shows that when examining the relation between brain activity and behavior with low statistical power, if a spatially restricted set of correlations is found, researchers should be aware that this may not reflect the full distribution of correlational effects that would be observed with higher power (a point also made by Cremers et al., 2017). In general, small sample sizes increase the risk of type II errors (false negatives) in identifying the full set of regions that correlate with behavior, but would nevertheless identify some regions that would show “true” correlations with behavior if the sample size were sufficiently large. On the other hand, unique areas where activation correlated with accuracy also were identified in small subsamples, some being found in only 1 of 7 or 10 subsamples (See Figures S4 and S6). This indicates that Type I errors, that is, false positives, are likely to occur as well as Type II errors when statistical power is low. This result leads to the conclusion that with sample sizes in the range of those often used in fMRI studies (i.e., 20–30 participants), one cannot be confident that all of the regions appearing to correlate with individual differences in behavior are reliable, or that other regions have not been missed altogether.

Although our results cannot be used to provide a definitive answer to the question of how many participants one needs to obtain a robust and stable brain‐behavior correlation, it is interesting that with both the Dallas and HCP data sets a sample size of roughly 80 participants was sufficient to achieve stable correlation magnitudes and significant LVs using PLS. However, it is also the case that the sample size needed for any given experiment will depend on a number of factors, including the type of task that participants carry out and the analytic approach to be used, as well as the characteristics of the sample (e.g., young vs. older adults, patients with a specific disorder, etc.). The analyses reported here are limited to the effects of sample size in healthy adults, removing the effects of age, and although the results generalize across the two experiments used here it is not clear how well our findings would apply to studies using patient populations, or different tasks. For example, the working memory and relational tasks that we used here are typical examples of an externally driven task in which stimuli are presented visually and participants are required to make a judgment about a stimulus property. Other types of task rely on cognitive processes that are internally driven, such as autobiographical memory retrieval or social/emotional decisions, and it is unknown whether the results reported here would characterize internally driven kinds of tasks. An additional limitation of this work is that for our aim of examining the effect of sample size on brain‐behavior correlations we were limited in the task data that could be used from the HCP study. A number of other tasks with in‐scanner performance measures are included as part of the HCP release, including social cognition tasks, but are either prone to ceiling effects in performance and/or have a restricted range of performance values, making them ill‐suited to the study of individual differences in brain‐behavior relations. We also note that the results reported here with whole brain analyses might not be applicable to brain‐behavior correlations measured with pre‐defined ROIs, whether these are defined anatomically or functionally. Future work will be needed to determine adequate sample sizes across a broad range of cognitive tasks and different ways of extracting brain activity.

Finally, Turner et al (Turner et al., 2018; Turner, Santander, Paul, Barbey, & Miller, 2019) have discussed the difficulties in recommending any specific sample size that would be suitable across all experimental studies, and have shown that within‐subject and between‐subject variability impact replicability, in addition to sample size and amount of per‐participant data that are collected. These authors have recommended a change in the methodological conventions that fMRI researchers use in their publications, such that it becomes standard practice to report “variables including replicability, as well as within‐participant and between‐participant variability” (Turner et al., 2019). Given these issues, as well as those surrounding power estimates, patient samples, and the host of other variables that might be involved in any fMRI experiment, we agree that attention should be paid to all of these variables when designing one's experiment and when reporting the results, including sample size and estimated power (Durnez et al., 2016; Poldrack et al., 2017). In addition, full reporting of such variables as power and effect size, as recommended in the framework compiled by the OHBM Committee on Best Practices in Data Analysis and Sharing (COBIDAS, Nichols et al., 2017), would be helpful in allowing readers to estimate how replicable any one experimental result is likely to be. Regardless, we emphasize that sample sizes of 20–30 participants are likely to be inadequate for identifying reproducible voxel‐wise correlations between behavior and brain activity in many, if not most, cognitive fMRI experiments.

In conclusion, we have presented evidence from two human fMRI data sets supporting the idea that small sample sizes can be particularly troublesome for brain‐behavior correlations. These results are consistent with previously reported simulation studies, but also provide novel experimental evidence from two independent data sets of the importance of sample size in obtaining stable results. An important aspect of our results is that multivariate approaches, such as the PLS approach used here, are not only more sensitive than a univariate approach to task‐related activations but also produce more extensive and consistent correlations between brain activity and behavior. This greater sensitivity might provide some benefit with smaller sample sizes, but should not be used as a substitute for giving full consideration to estimating a sufficient sample size when planning one's experiment. We hope that researchers will be able to use the results of this study to guide them in planning experiments and choosing a sample size appropriate to their scientific question, and to help evaluate the likelihood of true brain‐behavior effects reported in published work of varying sample sizes. Multicenter studies and large collaborative fMRI efforts, such as the HCP data set utilized here, are becoming more common and will mitigate the problem of small sample sizes for some types of research question. Nevertheless, it likely will still be the case for some time to come that many cognitive neuroscience experiments probing the neural correlates of specific cognitive processes will be carried out in single laboratories and researchers will need to ensure that issues of sample size and power are adequately addressed when brain‐behavior correlations are employed.

## CONFLICT OF INTEREST

The authors declare no conflicts of interest.

## Supporting information


**Appendix** **S1:** Supplementary Information

## Data Availability

The HCP data are freely available at http://humanconnectome.org/data/. The Dallas data are available from Dr. Kennedy upon reasonable request (kristen.kennedy1@utdallas.edu).
