# Supplementary material for: Influence of sample size and analytic approach on stability and interpretation of brain‐behavior correlations in task‐related fMRI data
Source: Hum Brain Mapp. 2020 Sep 30;42(1):204–19. doi: 10.1002/hbm.25217 (PMC7721240; doi:10.1002/hbm.25217)
Supplement: Supplementary file 1 — Appendix S1: Supplementary Information [file HBM-42-204-s001.pdf]

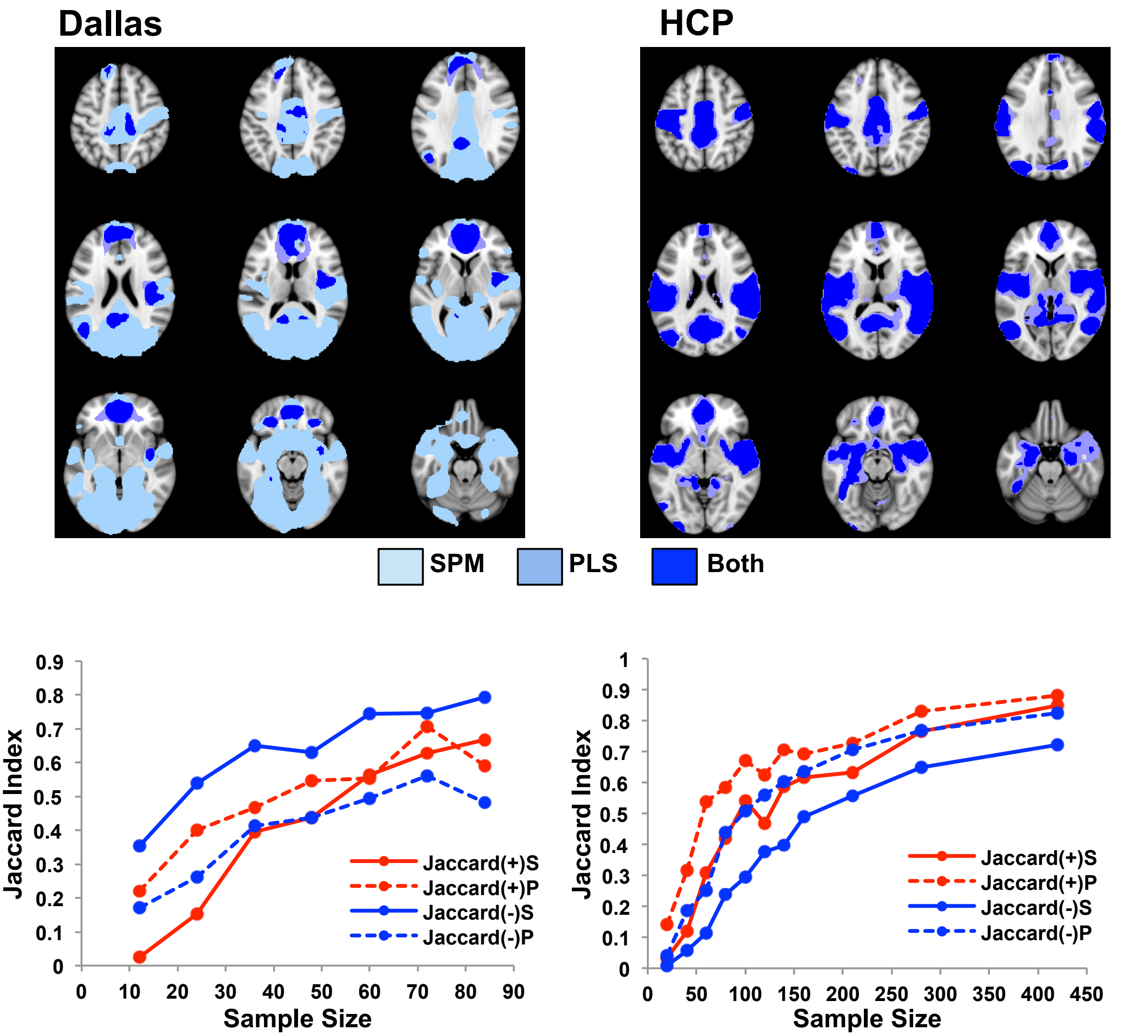

Supplemental Figure 1. The spatial maps and Jaccard indices are shown in blue for the task contrast of 0-back > 2,3,4-back ("negative" task effect, Dallas) and control > relational task (negative task effect, HCP). Jaccard indices for the reverse "positive" task effects (shown in red) are shown for comparison (also see Figures 1 and 5 in the main text). The "S" and "P" refer to SPM and PLS, and (+) and (-) refer to positive and negative task effects, respectively. Note that SPM identifies occipital regions with more activity during 0-back whereas PLS shows more activaton in occipital areas during 2,3,4-back (see Figure 1). However, despite some overlap, PLS finds peak activation in inferior occipital gyrus and peak deactivation found with SPM is in the lingual gyrus. This seeming contradiction could occur because weights for each condition are allowed to vary in PLS but not SPM, which could account for somewhat different results. Also, differences can be found across approaches because PLS attempts to find voxels that vary together across the whole brain, whereas SPM carries out statistics for each voxel.

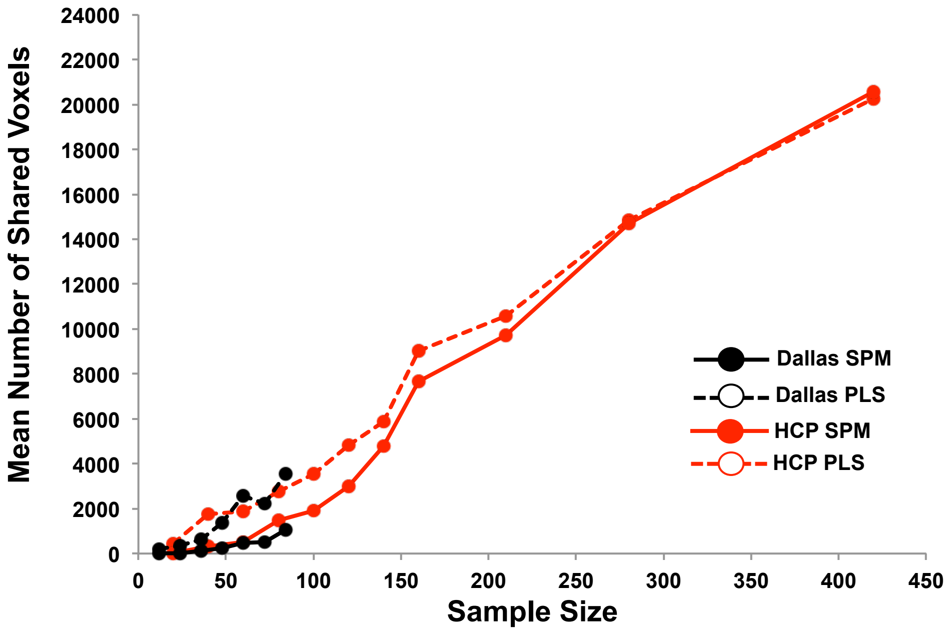

Supplemental Figure 2. This graph shows the number of above-threshold positively correlated voxels that are shared across pairs of images, averaged for each sample size, in the accuracy analyses using SPM and PLS. PLS identifies more shared voxels than SPM in both datasets. In addition, up to ~80 participants the curves are very similar for the Dallas and HCP datasets.

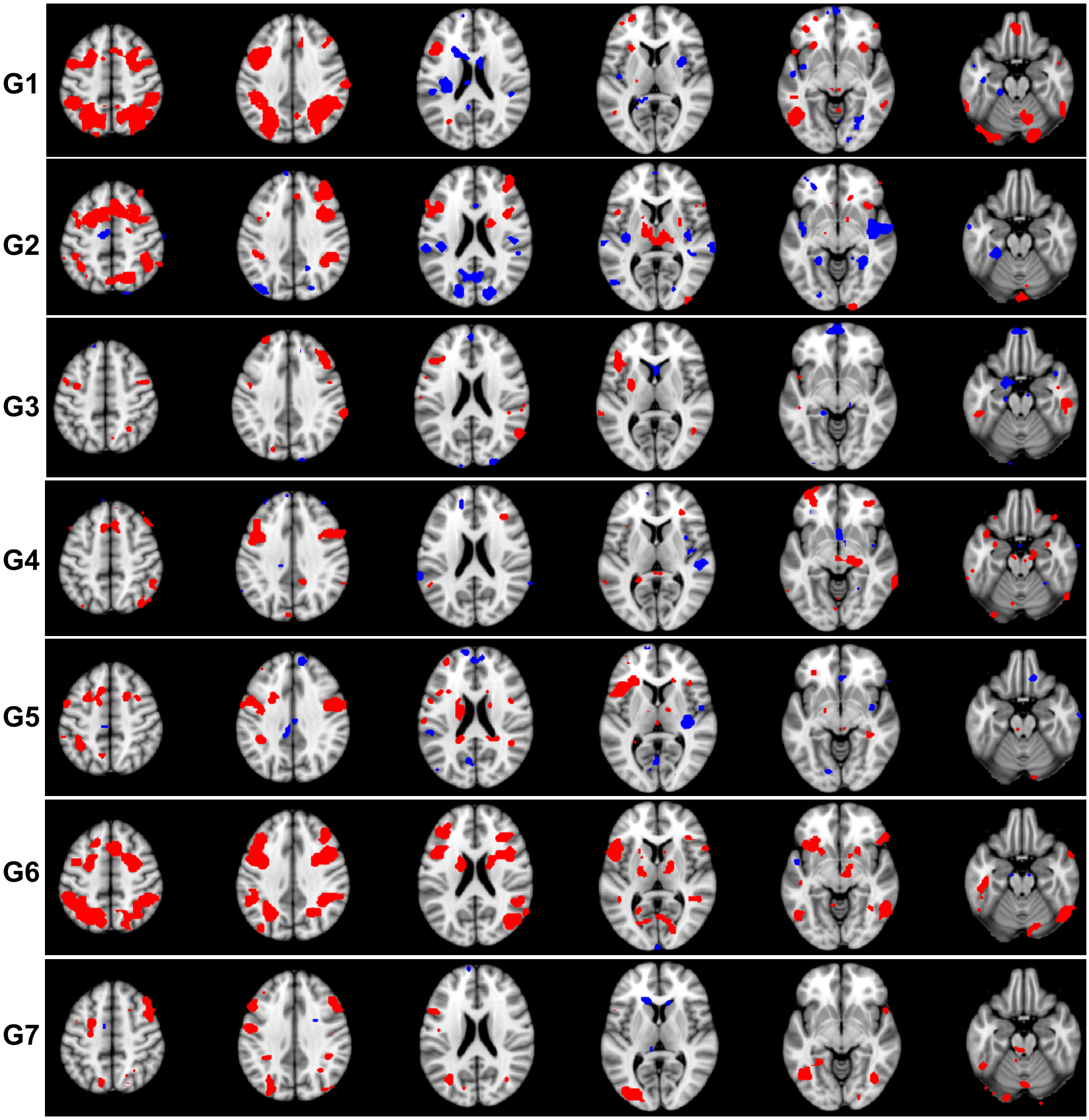

Supplemental Figure 3. Each of the 7 maps for the 24-participant subsample size is shown for the PLS analyses of the Dallas dataset (BSR > 3). Red areas indicate voxels where more activity during the working memory tasks was correlated with better accuracy, and blue voxels indicate voxels where less activity was correlated with better accuracy. Some areas, such as subcortical regions, are above threshold in only some of the maps, indicating variability across maps with this small sample size. G1-G7 refers to the 7 groups with 24 participants each.

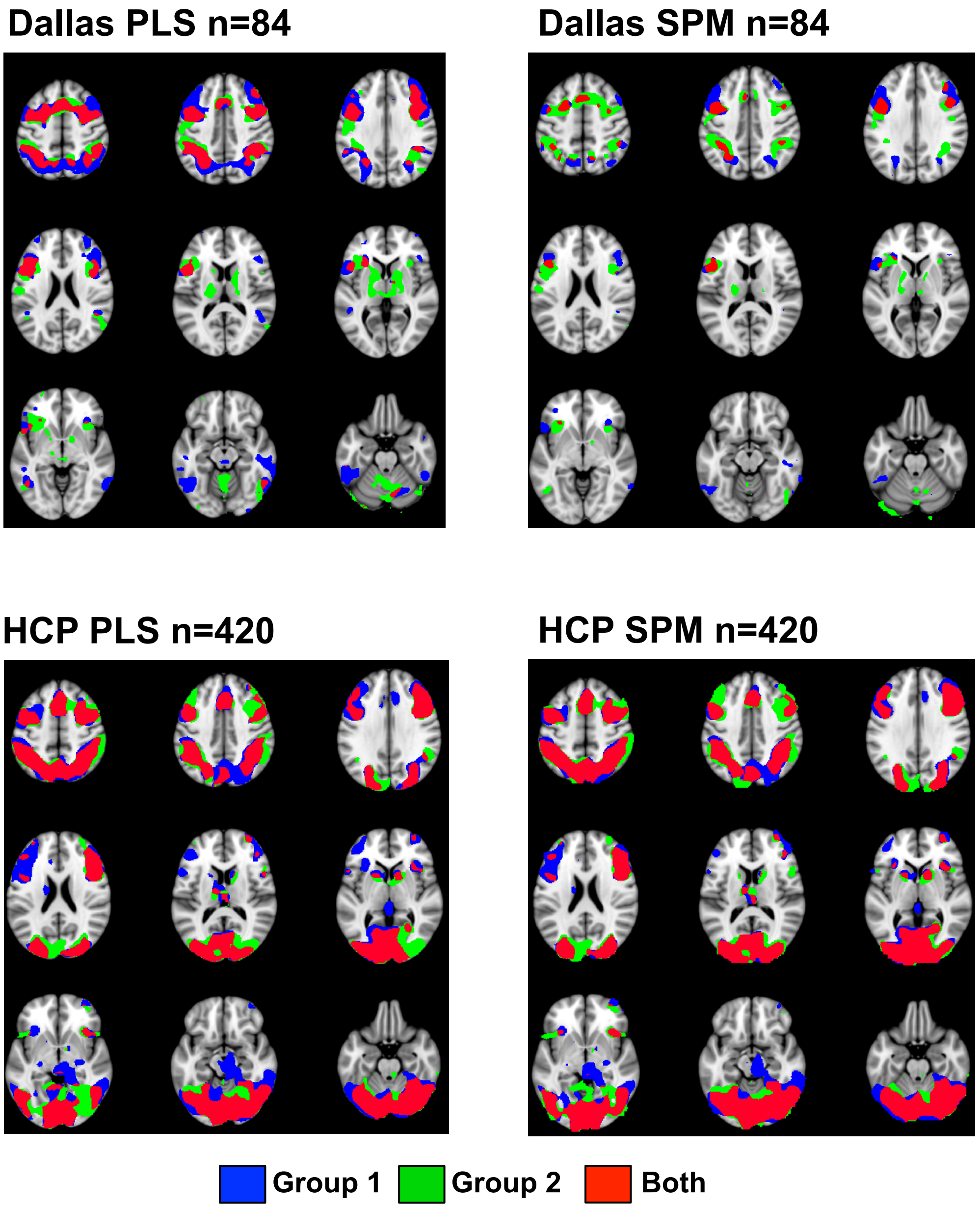

Supplemental Figure 4. Shown here are the spatial maps from the accuracy analyses for the largest subsamples in each dataset. The coloured voxels indicate the unique voxels for the two subsamples (blue for Group 1 and green for Group 2), and the common areas across both subsamples (red). Even with the much larger sample size of 420 in the HCP dataset the two spatial maps show some differences. Thresholds used to make this figure were t/BSR > 3 for Dallas and t/BSR > 4 for HCP.

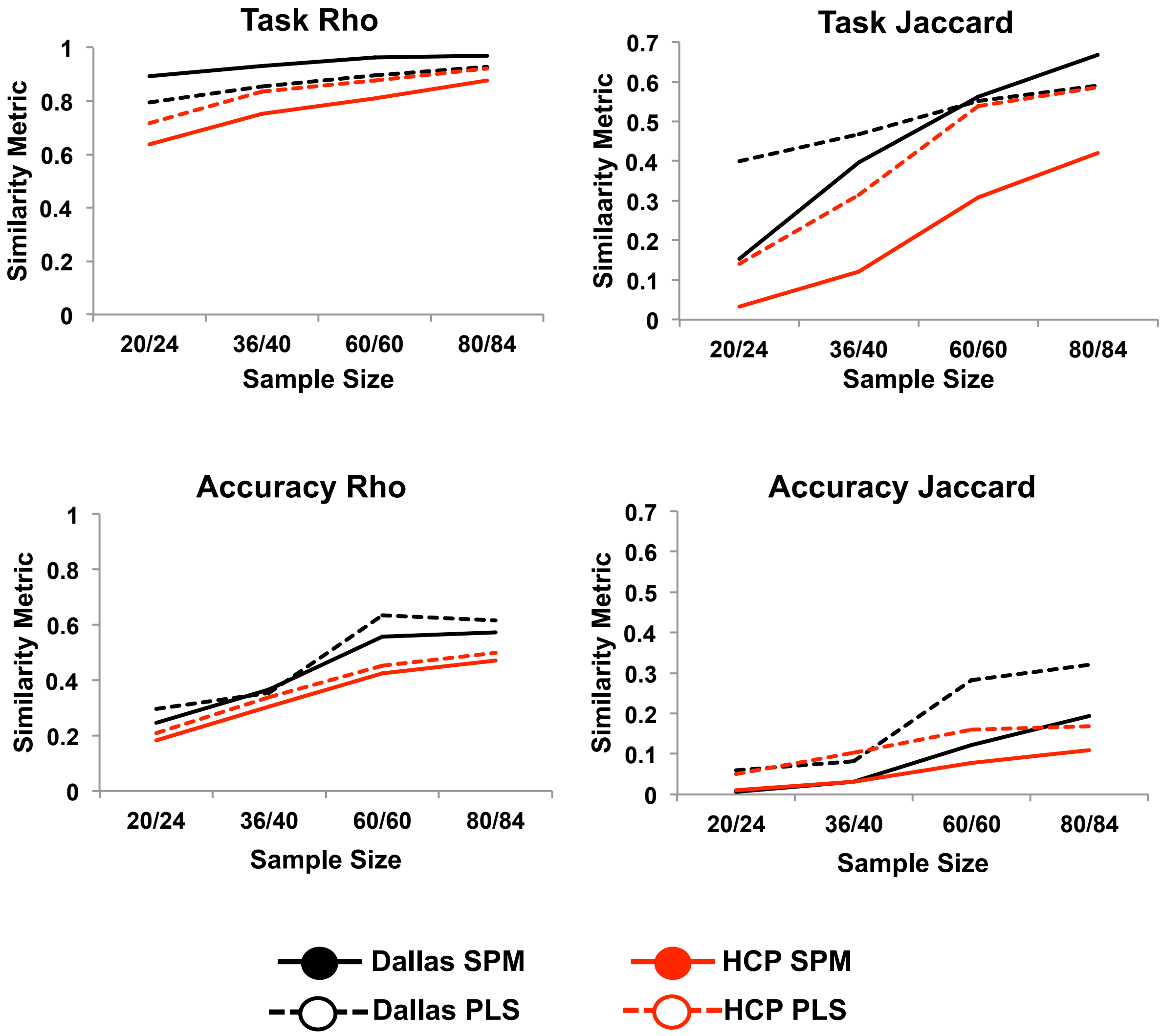

Supplemental Figure 5. Comparison of results for Dallas and HCP datasets from the task and behavior analyses for 4 of the sample sizes that are similar across datasets (e.g., 24 for Dallas and 20 for HCP). PLS (dashed lines) shows somewhat higher values than SPM (solid lines), particularly for Jaccard values. Dallas values also tend to be higher than those from the HCP dataset.

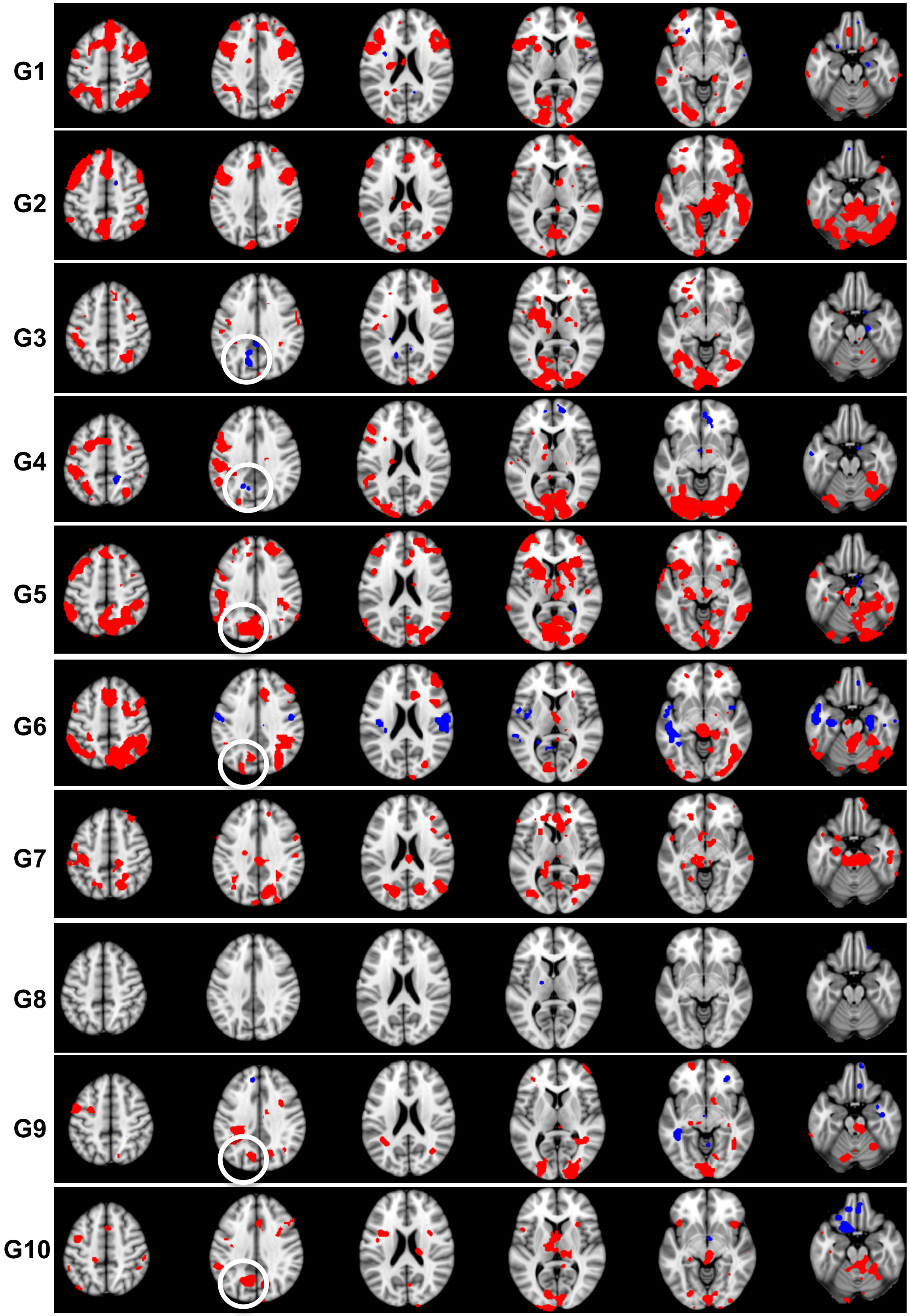
G1
G2
G3
G4
G5
G6
G7
G8
G9
G10

Supplemental Figure 6. Each of the 10 maps for the 20-participant subsample size is shown for the PLS analyses of the HCP dataset (BSR > 3). G1-G10 refers to the 10 groups with 20 participants each. Red areas indicate voxels where more activity during the relational task was correlated with better accuracy, and blue voxels indicate voxels where less activity was correlated with better accuracy. Some areas, such as subcortical regions, are above threshold in only some of the maps, indicating considerable variability across maps with this small sample size. In particular, G8 shows very few correlations between activity and performance. The white circles highlight an area of left medial parietal cortex where 4 subsamples had positive correlations with accuracy and two showed negative correlations.
